# Diagnosis of feline filariasis assisted by a novel semi-automated microfluidic device in combination with high resolution melting real-time PCR

**DOI:** 10.1186/s13071-019-3421-z

**Published:** 2019-04-08

**Authors:** Achinya Phuakrod, Witsaroot Sripumkhai, Wutthinan Jeamsaksiri, Pattaraluck Pattamang, Ekachai Juntasaro, Therdthai Thienthong, Suporn Foongladda, Paul J. Brindley, Sirichit Wongkamchai

**Affiliations:** 1 Department of Microbiology, Faculty of Medicine Siriraj Hospital, Mahidol University, Bangkok, Thailand; 2 Thai Microelectronic Center, National Electronics and Computer Technology Center, Thailand Science Park, Pathumthani, Thailand; 3 Department of Mechanical and Process Engineering, The Sirindhorn International Thai-German Graduate School of Engineering, King Mongkut’s University of Technology North Bangkok, Bangkok, Thailand; 4 Department of Microbiology, Immunology & Tropical Medicine & Research Center for Neglected Diseases of Poverty, School of Medicine & Health Sciences, George Washington University, Washington, DC USA; 5 Department of Parasitology, Faculty of Medicine Siriraj Hospital, Mahidol University, Bangkok, Thailand

**Keywords:** Filariasis, Microfluidics, Detection of microfilariae in blood, HRM real-time PCR

## Abstract

**Background:**

The diagnosis of filariasis traditionally relies on the detection of circulating microfilariae (mf) using Giemsa-stained thick blood smears. This approach has several limitations. We developed a semi-automated microfluidic device to improve and simplify the detection of filarial nematodes.

**Methods:**

The efficiency and repeatability of the microfluidic device was evaluated. Human EDTA blood samples were ‘spiked’ with *B. malayi* mf at high, moderate, and low levels, and subsequently tested 10 times. The device was also used for a field survey of feline filariasis in 383 domesticated cats in an area of Narathiwat Province, Thailand, the endemic area of *Brugia malayi* infection.

**Results:**

In the control blood arbitrarily spiked with mf, the high level, moderate level and low level mf-positive controls yielded coefficient variation (CV) values of 4.44, 4.16 and 4.66%, respectively, at the optimized flow rate of 6 µl/min. During the field survey of feline filariasis in Narathiwat Province, the device detected mf in the blood of 34 of 383 cats (8.9%) whereas mf were detected in 28 (7.3%) cats using the blood smear test. Genomic DNA was extracted from mf trapped in the device after which high-resolution melting (HRM) real-time PCR assay was carried out, which enabled the simultaneous diagnosis of filarial species. Among the 34 mf-positive samples, 12 were identified as *B. malayi*, 15 as *Dirofilaria immitis* and 7 as| *D. repens*.

**Conclusions:**

We developed a semi-automated microfluidic device to detect mf of filarial parasites that could be used to diagnose lymphatic filariasis in human populations. This novel device facilitates rapid, higher-throughput detection and identification of infection with filariae in blood samples.

## Background

The filariae are a group of parasitic nematodes that belong to the family Onchocercidae. Filarial species that parasitize cats and dogs including *Dirofilaria immitis*, *D*. *repens*, *Brugia malayi* and *B. pahangi* are of worldwide zoonotic and veterinary significance and are recognized as emergent human pathogens [1–4]. Accurate and prompt diagnosis is essential for the management of filarial infection at the individual level and for disease control in populations living in endemic regions. Traditionally, the diagnosis of filariasishas relied on the detection of microfilariae in peripheral blood, using microscopical examination of stained, thick blood smears. According to the World Health Organization (WHO), the drying step of the thick blood smear may require 12 hours in the dry season while 24–48 hours may be needed in the high humidity during the rainy season [5].

Over the past decade, microfluidic technologies, i.e. the precise control of fluids and samples at sub-millimeter scale, adapted for the detection of pathogens in mammalian bodily fluids, have emerged as powerful, facile tools. The microfluidic approach has been applied for the detection of diverse pathogens including blood stages of malarial parasites [6–9]. The premise of microfluidic technologies is the manipulation and analysis of fluids within micro-channels. The construction of the device is accomplished using microfabrication processes, such as photolithography (PL), deep reactive ion etching (DRI), replica casting, silicon (Si) molds, and others [10]. Microfluidic-based platforms offer advantages in comparison to thick blood smear staining technique, including speed, cost, portability, high throughput and automation [9]. Moreover, microfluidic devices require and consume only minute amounts of samples and reagents, which minimizes waste and expense, and they offer the unique physical advantage of microscale fluid flow [10]. Microfluidic technologies have also been used in research on other parasitic nematodes, including the deployment of a chip device that integrates microfluidics and electrophysiology to provide real-time records of the nano-scale electrical signals emitted by nematode muscles and neurons [11], and a microfluidic electropharyngeograms to screen anthelmintic candidates and investigate feeding behaviors by nematodes for the discovery of novel anthelmintics [12].

In this study, we report a novel, semi-automated microfluidic device that integrates real-time and high resolution melting PCR for the simultaneous detection and differentiation of species of filariae, in the blood of domesticated cats naturally infected with species of *Brugia* and *Dirofilaria*.

## Methods

### Study samples

**Fig. 1:**
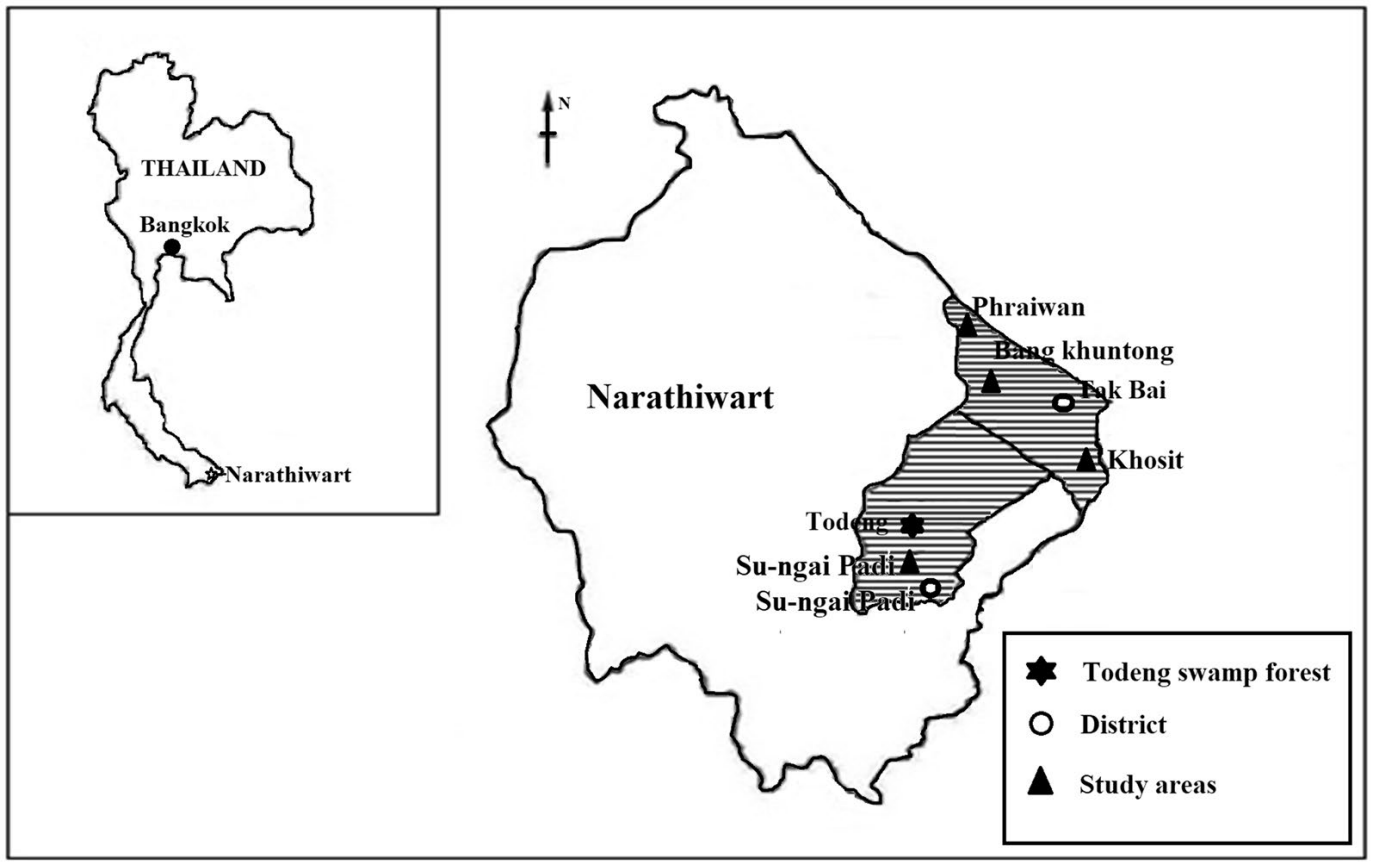
Map of Narathiwat Province, Thailand showing the study areas and brugian filariasis endemic areas

To study the efficiency and reproducibility of the semi-automated microfluidic device, three groups of samples were prepared by spiking microfilariae (mf) of *B. malayi* into EDTA blood obtained from a healthy subject. For the survey of feline filariasis in Narathiwat, blood was collected from 383 cats residing in Su-ngai Padi and Tak Bai districts of Narathiwat Province (Fig. 1).

### Detection of microfilariae using a semi-automated microfluidic device

#### Microfluidic chip design and fabrication

**Fig. 2:**
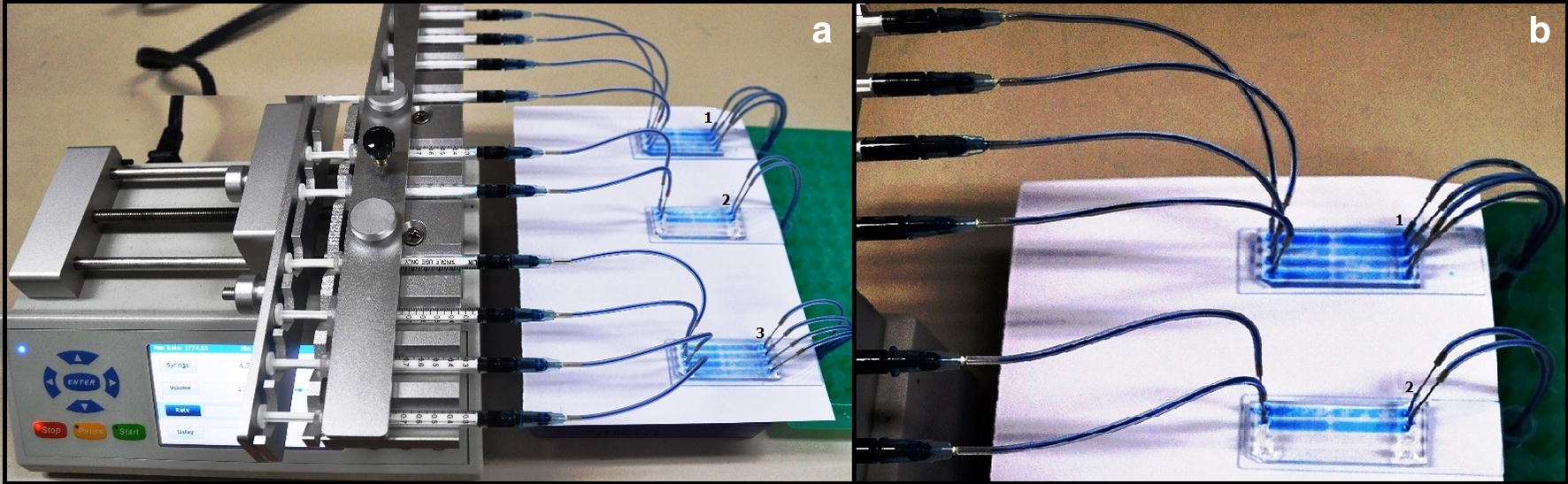
The microfluidic device for detection for diagnosis of the microfilarial stage of filarial parasites. The sample injector consists of an infusion pump with an adaptor and ten syringes with a 5 mm long blunt ended needle connected to the inlet of the microfluidic chips (1, 2, 3) using silicone tubing. **a** Microfluidic device. **b** Microfluidic chips

The four-channeled microfluidic chip used in this study was adapted from the microfluidic chip reported earlier [13]. In brief, the microfluidic chip was fabricated from polydimethylsiloxane (PDMS) with patterned Photoresist on a silicon wafer [14]. For the present investigation, the pattern features were created on a Si wafer through PL and DRIE processes. The resulting Si master is a molding template for casting PDMS. A 10:1 mixture of PDMS pre-polymer and curing agent was cast with the Si master and the polymer cured at 60 °C for 3 h. Subsequently, the cured polymer from the PDMS replica was removed from the master, and cut to the required shape using a sharp cutter. Inlet and outlet ports were constructed by punching holes through the PDMS chip. The PDMS replica was sealed to a glass slide after the interface bonding process to promote the oxygen plasma process. Silicone tubing was inserted into the holes and sealed with epoxy resin. Figure 2 depicts the microfluidic system, which consists of an infusion pump with an adapter and a 1 ml syringe with a bespoke 5 mm long needle with a blunt end that serves as the injector port for the samples. Up to 10 samples can be processed simultaneously.

#### Optimization of flow rate

To optimize the flow rate, testing of the microfluidic device was performed using the same set of mf-positive blood samples. The average number of mf in the blood samples was 60 mf per 50 µl of sample. The device was tested with flow rates of 6, 8 and 10 µl/min. Duplicate flow rates were performed. The run time and the numbers of the trapped mf in the chip were recorded. The choice of optimal operating flow rate was established based on duration of the run and numbers of trapped mf.

#### Sample preparation and mf detection by the microfluidic device

**Fig. 3:**
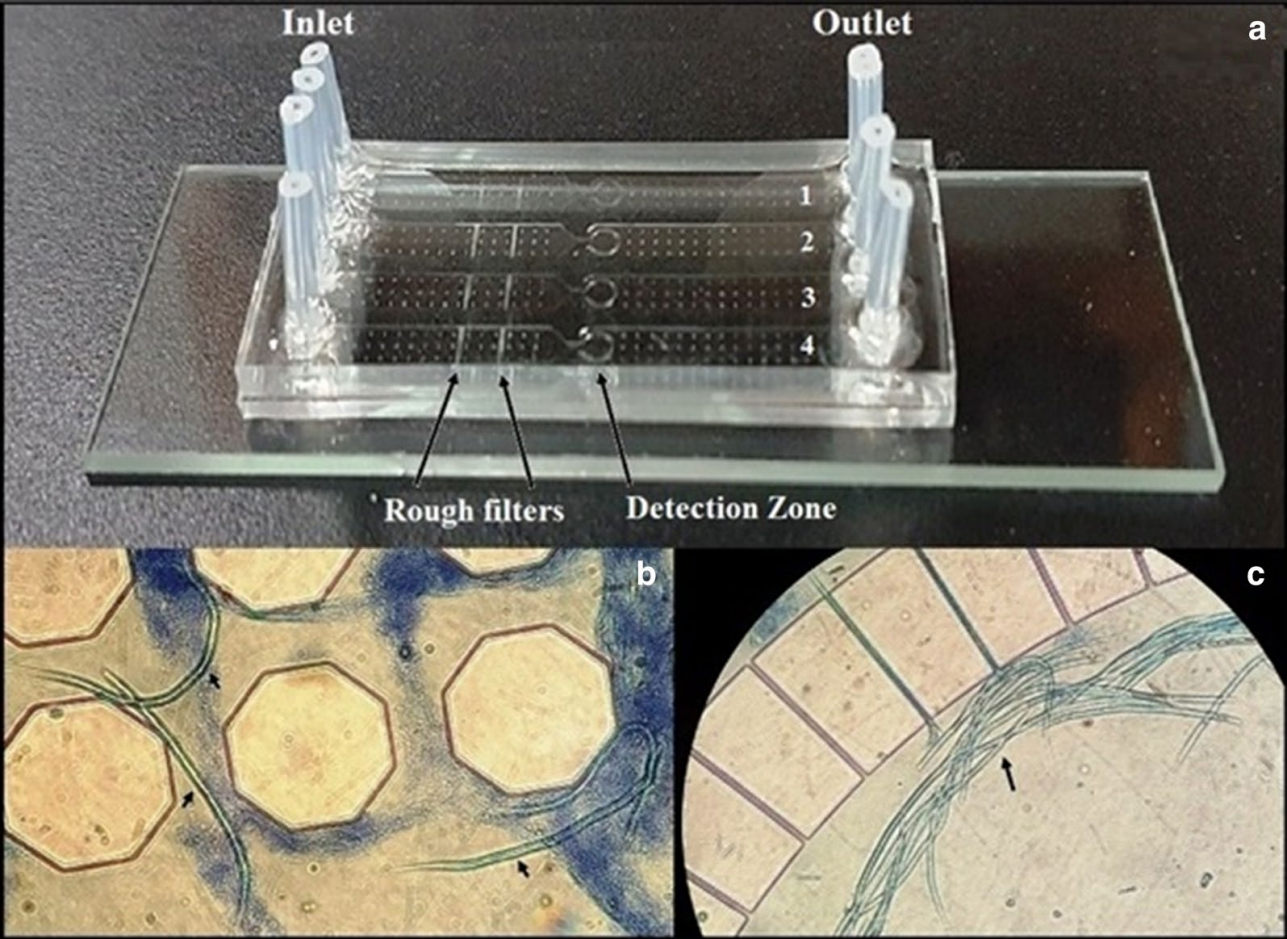
Microfluidic chip for detecting microfilariae of filarial parasites. **a** The chip contains four testing channels; each channel consists of four components: the inlet, rough filters, detection zone and outlet from left to right. **b** Photomicrograph of microfilariae of lymphatic filariae trapped in the rough filter area. **c** Photomicrograph of microfilariae of lymphatic filariae trapped in the detection zone of the device (10× magnification)

Before testing, 50 µl of EDTA-treated blood was suspended into 150 µl of the specifically-formulated lysis buffer A, which contained a Triton X-100 and bromophenol blue, mixed and incubated at room temperature for 10 min. The prepared sample was drawn, followed by 15 µl of buffer B containing a chaotropic salt and PBS, into a 1 ml syringe with a specially designed 5 mm long blunt end needle that connects the syringe to the adapter and to the inlet port. The pump was started and the sample solution was introduced into the microfluidic device via the inlet port. The microfilariae were trapped in the microfluidic chip while the remaining solution exited the microfluidic chip via the outlet port and into the waste tube. The trapped mf were inspected by light microscopy at 10× magnification. The semi-automated microfluidic device and the microfluidic chip are shown in Figs. 2, 3.

#### Evaluation of the efficiency and repeatability of the microfluidic device

To evaluate the efficiency of the microfluidic device, blood donated by a healthy human was collected in EDTA and subsequently ‘spiked’ with microfilariae of *B. malayi* to prepare high level (average 30 mf/50 µl), moderate level (average 15.5 mf/50 µl) and low level (average 7.5 mf/50 µl) mf-positive control blood samples. To study the intra-assay variation, 10 replicates of each of these three mf-positive controls were tested in the microfluidic device, which was operated at a flow rate of 6 µl/min. The mean, SD, % CV and range of both the trapped mf and leaked mf were calculated for intra and inter assay variation. Inter assay variation was calculated from the average mf/sample on each of five consecutive days.

### Microscopic detection of microfilariae (mf) using Giemsa staining

Giemsa staining of thick blood smears was performed according to the standard WHO procedure [5]. Briefly, 50 µl of EDTA blood was dropped/spotted onto a glass slide, then smeared in a circular shape using the edge of a fresh microscope slide, and then the smeared slide was air-dried overnight (15–18 h) at room temperature. Subsequently, the slide was immersed in water followed by staining for 45–60 min with a freshly prepared working solution of Giemsa stain. After air-drying, the slides were examined under a microscope (40×) for mf by skilled technicians.

### Field survey of feline filariasis

Samples of blood from a total of 383 domesticated cats were obtained from an annual survey of filarial infection in the Su-ngai Padi and Tak Bai districts of Narathiwat Province, the endemic areas of brugian filariasis, conducted by the Filariasis Project, Pikhunthong Royal Development Study Center, Narathiwat, Thailand. A single thick smear slide was prepared from blood obtained by ear prick from each cat. Additional blood was collected from each cat and dispensed into heparinized capillary tubes. The thick blood smear staining technique was performed in the laboratory of the Filarial Project, Phikhunthong Royal project, Narathiwat Province. Microfluidic device testing was performed in the laboratory of the Department of Parasitology, Faculty of Medicine Siriraj Hospital, Mahidol University, Bangkok. All of the study cat blood samples, which were tested for microfilarial detection using the microfluidic device, were blindly performed.

### Species identification of the trapped microfilariae (mf) using HRM real-time PCR analysis

#### Extraction of filarial DNA from trapped mf in the microfluidic chip

To identify species of the trapped mf in the microfluidic chips, 100 µl Tris-EDTA buffer was dispensed into the chip via the inlet port. The chip was placed on a hot plate at 56 °C for 15 min, after which the solution containing the trapped mf was withdrawn through the outlet port and transferred to a 1.5 ml Eppendorf tube. The tube was subjected to centrifugation at 15,520× *g* for 10 min. The supernatant was discarded, and DNA was extracted from the pelleted material using the Roche high pure PCR template preparation kit, according to the manufacturer’s instructions (Roche Diagnostics GmbH, Penzberg, Germany). DNA concentration was determined using a Nano Drop (Thermo Fisher Scientific) after which the DNA was employed as the template for HRM real-time PCR analysis. The DNA concentration of the 34 mf-positive samples is range between 5.9–11.4 ng per µl.

#### HRM real-time PCR assay

**Fig. 4:**
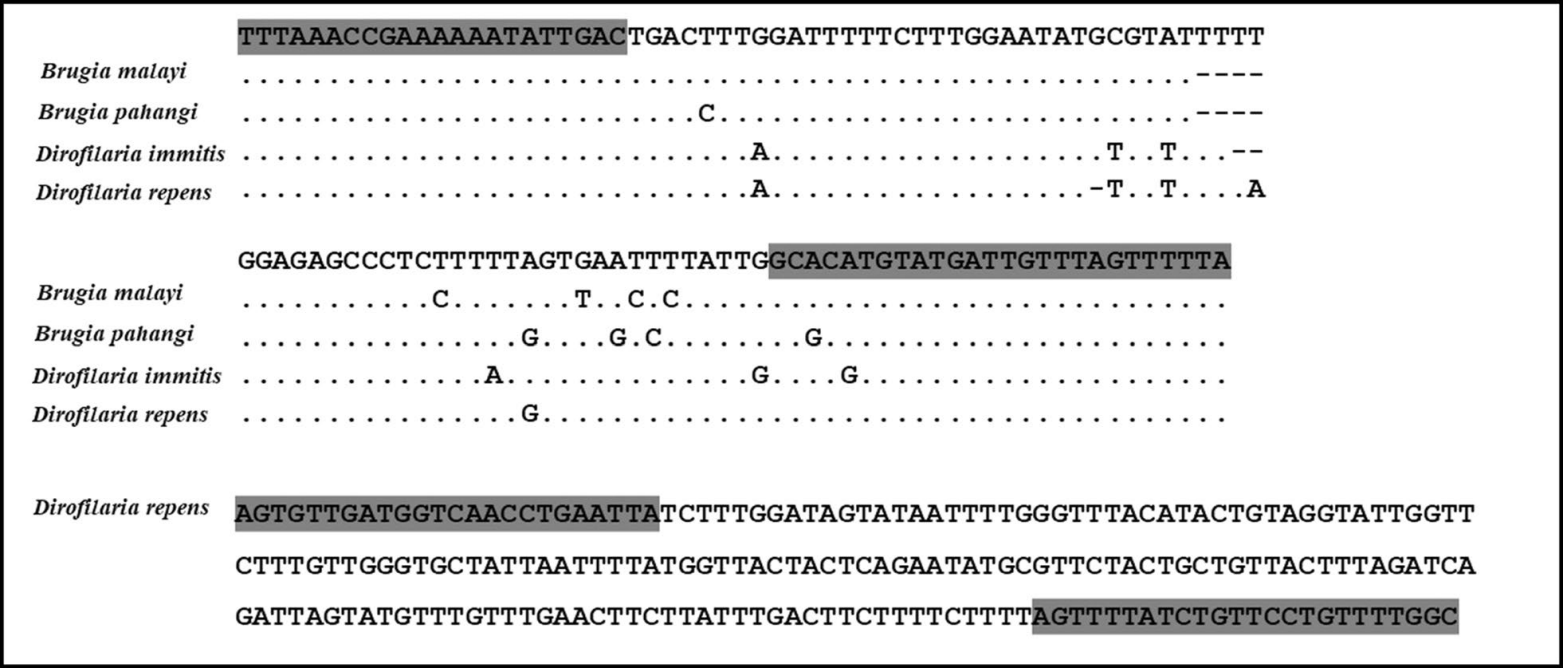
Alignment of nucleotide sequences of the partial mitochondrial *12S* rRNA gene of *B. malayi*, *B. pahangi*, *D. immitis* and *D. repens*, as well as alignment of the *cox*1 gene of *D. repens*. Dots indicate identity and dashes indicate deletion from the above consensus sequence. Gray areas indicate PCR primer sequences

The HRM real-time PCR assay was performed on a Light-Cycler LC480 instrument (Roche, Penzberg, Germany) with primers reported previously [15]. To identify *B. malayi*, *B. pahangi*, *D. immitis* and *D. repens*, DNA of these four filarial species were used as positive controls (Fig. 4). Nuclease-free water replaced the DNA template for the negative control. Reaction conditions included an activation step at 95 °C for 5 min followed by a 40-step amplification of 10 s at 95 °C, 10 s at 58 °C and 10 s at 72 °C. Subsequently, the products were heated to 95 °C for 1 min and then cooled to 40 °C for 1 min, followed by HRM from 65 to 95 °C. The LightCycler 480 gene scanning software (Roche) was employed to construct melting curves, which were normalized, temperature-shifted and converted to difference plots. Little difference in melting temperature (Tm’s) (< 0.5 °C) was found between melting curves for *D. immitis* and *D. repens* and hence it was necessary to distinguish these two species of *Dirofilaria* by PCR. This was done targeting the cytochrome oxidase subunit 1 (*cox*1) gene, using the following primers specific for the *cox*1 gene of *D. repens*: 5′-AGT GTT GAT GGT CAA CCT GAA TTA-3′ and 5′-GCC AAA ACA GGA ACA GAT AAA ACT-3′ [16]. The HRM was performed as previously described [16].

## Results

### Microfluidic chip design and fabrication

The fabricated microfluidic chip consists of four parallel microfluidic channels, each of which contains an inlet and outlet, and an enclosed chamber in the middle of each channel (Figs. 2, 3). The height of the microfluidic channels is 50 μm and the width of the inlet and outlet channels is 200 μm. Each enclosed area has a diameter of 2.0 mm and contains an integrated flow-through microfilter. The microfilter has a distance of 3 μm between rectangular pillars.

### Flow rate optimization

The number of trapped mf and elapsed time were recorded at specified flow rates. The elapsed time at a flow rate of 6 µl/min was 37 min while the elapsed times at flow rates of 8 µl/min and 10 µl/min were 25 and 17 min, respectively. The number of trapped mf at 6 µl/min was 59.5, while 45 mf were trapped at 8 µl/min and 28.5 mf were captured at 10 µl/min (Fig. 3). The flow rate of 6 µl/min was chosen since it trapped the highest amount of mf.

### Microfluidic device testing time per sample

The operator required 15 min to prepare 10 samples, 37 min to perform the testing, and 5 min for mf observation under the microscope. Therefore, approximately 5.7 min were needed to complete one sample.

### Evaluation of the efficiency and repeatability of the microfluidic device

To evaluate intra-assay and inter-assay variation, the mean ± SD of the trapped mf and the mean ± SD and % CV of the mf from the outlet (leaked mf) of blood samples with high, moderate and low mf levels were recorded (Table 1).

**Table 1:** The efficacy and repeatability of the microfluidic device in the laboratory

Microfilariae level/50 µl blood	Number of microfilariae							
	Intra-assay variation				Inter-assay variation			
	Trapped mf (mean ± SD)	CV (%)	Leaked mf (mean ± SD)	CV (%)	Trapped mf (mean ± SD)	CV (%)	Leaked mf (mean ± SD)	CV (%)
High (mean 30)	27.22 ± 2.94	10.8	1.25 ± 0.83	66.4	31 ± 1.22	4.44	1.61 ± 0.55	34.16
Moderate (mean 15.5)	15.44 ± 1.3	8.42	1.56 ± 1.2	76.9	15.6 ± 0.89	4.16	1.4 ± 0.55	32.49
Low (mean 7.5)	5.25 ± 0.5	9.38	0.5 ± 0.58	94.6	5.8 ± 0.45	4.66	0.4 ± 0.55	45.8

*Abbreviations:* CV, coefficient of variation; mf, microfilariae; SD, standard deviation

### Microfilariae detection in cat blood samples by the microfluidic device

Microfilariae were detected in the blood of 34 of 383 cats (8.9%) using the microfluidic device, whereas mf were detected in blood of 28 cats of these 383 cats (7.3%) by the Giemsa-stained thick blood smear technique. The species of the mf trapped in the microfluidic chamber were identified by real-time PCR with HRM analysis (Table 2).

**Table 2:** The prevalence of filarial detection in domestic cats in Narathiwat Province, Thailand by microfluidic device compared with the Giemsa staining method, showing species of the detected filariae

Method		No. of samples (%)	Species of mf		
Giemsa stain	Microfluidic chip		*B*. *malayi*	*D. immitis*	*D. repens*
+	+	28 (7.3)	9	13	6
−	+	6 (1.6)	3	2	1
+	−	0	0	0	0
−	−	349 (91.1)	–	–	–

*Abbreviations:* mf, microfilariae

### HRM real-time PCR assay

**Fig. 5:**
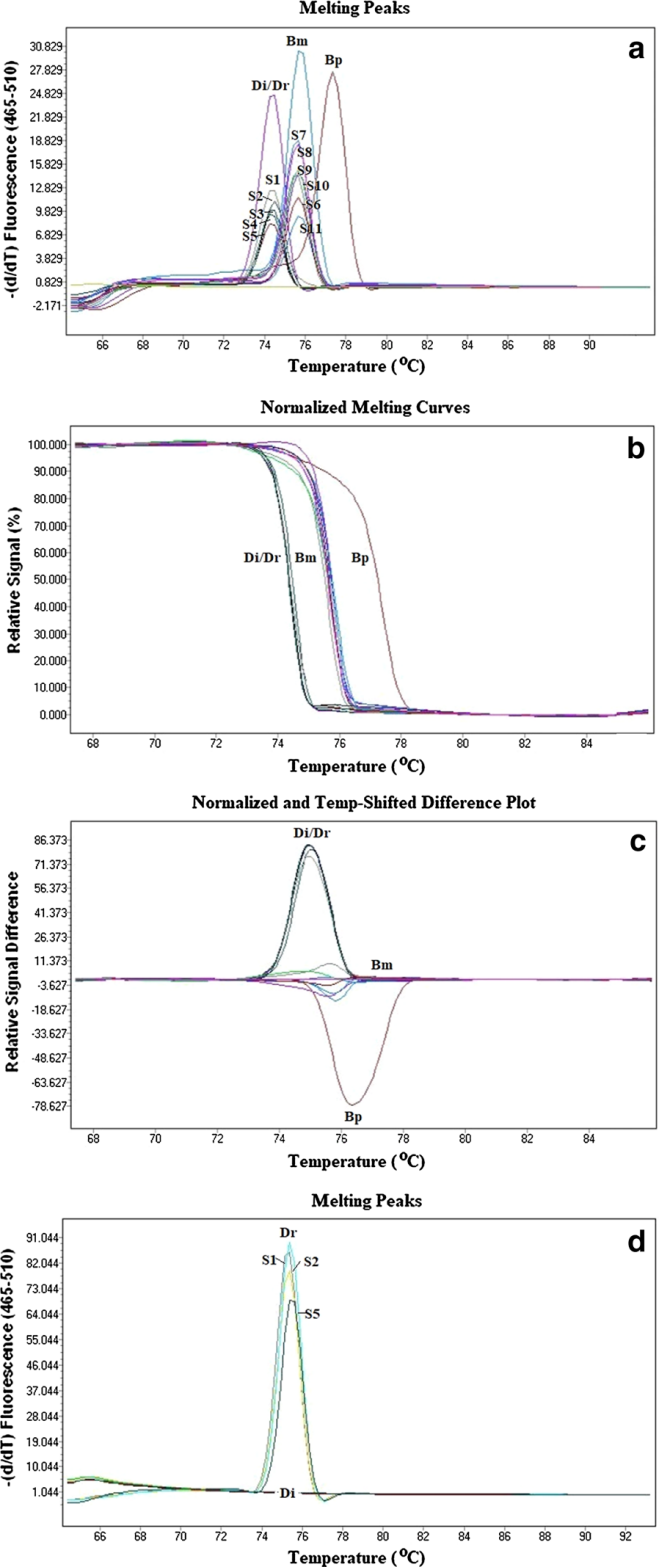
Melting peaks (**a**), normalized difference curves (**b**) and the normalized and temperature-shifted difference plot (**c**) of the amplified product of control species (*B. malayi*, *B. pahangi* and *D immitis/D. repens*) and S1-S11 representative DNAs from 34 mf-positive samples of cat blood and DNA from the 22 mf-positive samples using *cox*1 specific primers for *D. repens*, S1; S2; S5 represented the 22 mf-positive cat samples (**d**), as obtained with the LightCycler 480 gene scanning software. *Abbreviations*: Bm, *B. malayi*; Bp, *B. pahangi*; Di, *D immitis/D. repens*

Figure 5 presents a representative HRM finding with amplicons from mf-positive blood and the studied samples, including the melting curve analysis and the normalized and temperature-shifted difference plot. Using the primers of Wongkamchai et al. [15] the amplicons for the positive controls (Bm, *B. malayi*; Bp, *B. pahangi*; Di, *D. immitis* and Dr, *D. repens*) obtained from the Light Cycler 480 software, were recognized by the HRM assay at melting peaks (Tmʼs) of 75.6 ± 0.08 °C, 77.19 ± 0.12 °C, 74.07 ± 0.21 and 74.15 ± 0.16 °C, respectively. Twelve mf-positive cats were determined to be positive for *B. malayi* mf whereas 22 mf-positive cats were confirmed to be infected with either *D. immitis* or *D. repens*. Thereafter, the DNAs from these 22 mf-positive samples were investigated using the *cox*1 gene-specific primers for *D. repens* [16]. The amplicon from the *D. repens* positive control was recognized in the HRM assay at melting peak (Tmʼs) of 75.32 ± 0.12 °C whereas the findings were negative when tested with *D. immitis* and with negative control cat blood samples (Fig. 5). In total, 15 mf-positive cats were identified as infected with *D. immitis* and seven mf-positive cats with *D. repens*.

## Discussion

The analysis of diverse analytes using a single biochip has gained wide utility over the past decade due to its efficacy in the process of identification, diagnosis and discovery, especially for clinical samples [9]. We developed a semi-automated microfluidic device to detect microfilariae of filarial worms. Microfluidic channels within the device facilitated the sieve-like sorting of the microfluidic device. The flow control was achieved by the application of an infusion pump. For the pressure-driven flow used in microfluidics-based assays, it is important to determine the maximum flow rate that allows retention of mf within the chip. Improvements in assay speed attempted by raising the flow rate excessively may cause more mf to be forced through the filter to the outlet, thereby decreasing sensitivity and efficiency. We deployed a flow rate of 6 µl/min as a compromise rate because no mf were found in the outlet and the time spent for one run was not significantly longer than those obtained with higher flow rates.

Sample preparation on microfluidic devices is a continuing challenge and a necessary component of developing fully integrated and facile microfluidic diagnostic approaches [17]. When working with biological or clinical samples that are in limited supply, sample preparation remains a weak link in microfluidics chip assays [18]. In our study, lysis of the blood cells is necessary to overcome high concentrations of blood cell components, which can obstruct the flow. Triton X, a non-ionic detergent [19], was used as the lytic reagent in the sample buffer, and a chaotropic salt was included in the microfluidic chip to facilitate extraction of nucleic acids within the chip [20]. For the sample injection, we applied an adapter to the syringe pump that enabled testing of up to ten samples in a single run. The sample-injector was constructed to include a 1 mm syringe fitted with a blunt ended needle of the desired size. The commercially available needle is 7 cm (2.5 inches) in length bearing a standard, needle-sharp point. In place of the commercial needle, we inserted a short (5 mm), blunt-ended needle, which was simple to construct. This modification rendered the sample-injector safer for the operator to insert into the silicone tube, and also reduced the sample loading time. Another advantage of this bespoke device is the glass slide size of the chip. It includes four channels in concert with the clear background. The mean time per sample was 7.5 minutes. By using the device, a larger number of blood samples could be managed, and results could be obtained within an hour. Since 50 µl of EDTA-treated blood was used per sample, the detection limit (sensitivity) of our developed device was ≥ 20 mf in 1 ml of blood. Moreover, no false positive was found. The device can also be applied for the screening of lymphatic filariasis in human blood. The microfluidic device is portable and weighs three kilograms. However, it requires electrical power to operate, which may restrict its use in more remote settings. Fewer mf were detected using the thick blood smear staining technique compared to the microfluidic device. In an early report on the use of stained thick blood smears for parasitological diagnosis of filariasis, Southgate [21] compared the counting chamber technique with stained thick blood smear technique to detect microfilariae of *W. bancrofti* in human blood; all mf-positives by the counting chamber technique were positive by blood film staining technique whereas 77 mf-positive blood samples by counting chamber technique were negative by thick blood smear staining. Southgate concluded that the 77 mf-negative blood obtained from thick blood smear staining technique was due to the loss of mf from the slide during slide preparation [21]. Moreover, losses of microfilariae from blood films result from errors with slide preparation including the use of glass slides that are not clean, blood smears that are too thick, and importantly, insufficiently dried blood films [5, 22]. From our own experience with thick blood smears, human error resulting in false negative mf results is an important problem. This is because performing large numbers of blood samples is tedious, laborious and time consuming. The presence of stained red blood cells in the Giemsa stained smears causes eye fatigue and dizziness in many operators, and can reduce the efficacy and sensitivity of the assay.

Nevertheless, application of the microfluidic device for filarial detection in cats causes a concern since cats may be infected with both human and zoonotic filariae. Although the mf can be differentiated as sheathed or non-sheathed, the identity of the species may not be clear for non-sheathed filariae in cat blood. Moreover, it is particularly difficult to differentiate mf of *B. malayi* (human filaria) and *B. pahangi* (feline species) by Giemsa staining due to their similar morphology [23]. To obviate this concern, we integrated a HRM real-time PCR assay for species differentiation of the trapped mf. Advantages of this technique include that the initial step of DNA extraction can be performed within the chip, and the PCR with HRM analysis can be performed in a single tube using a single pair of primers with no specific probes required. The assay can be performed with sets as large as 384 samples in a single run and without the need for a downstream follow-up PCR step [24, 25]. Furthermore, the assay is sensitive and detects as little as a 19.4 pg genomic DNA/reaction for *B. malayi* and a 16.4 pg/reaction for *D. immitis* [13]. The filarial species in all 34 positive cat blood samples were identified satisfactorily.

To conclude, we have developed a semi-automated microfluidic device to detect mf of filarial parasites that could be used to diagnose lymphatic filariasis in human populations. Although there are numerous reports dealing with DNA-based detection of filariasis, including the rapid differentiation of *D. immitis* and *D. repens* [26, 27], our study is the first to link the microfluidic system with the HRM real-time PCR to achieve a reliable and cost-effective method for rapid identification of feline filarial parasites. This novel device facilitates rapid, higher-throughput detection and identification of infection with filariae in blood samples.

## Conclusions

We developed a high-throughput, semi-automated microfluidic device integrated with HRM real-time PCR assay for detection and identification of filarial worms in peripheral blood sampled from cats. The microfluidic device paired with the HRM real-time PCR assay enabled rapid simultaneous diagnosis of up to ten discrete blood samples. Device and constituent reagent patents are being sought in Thailand, Patent Request Number 1701001524.

## Abbreviations

Bm: *B. malayi*
Bp: *B. pahangi*
Di: *D immitis*
Dr: *D. repens*
partial mitochondrial *12S* rRNA gene: partial mitochondrial 12 subunits of ribosomal ribonucleic acid
*cox*1: cytochrome *c* oxidase subunit 1
HRM: high resolution melting
PCR: polymerase chain reaction
PL: photolithography
DRIE: deep reactive ion etching process
